# In vivo examination of healthy human skin after short‐time treatment with moisturizers using confocal Raman spectroscopy and optical coherence tomography: Preliminary observations

**DOI:** 10.1111/srt.13101

**Published:** 2021-09-23

**Authors:** Cristel Ruini, Benjamin Kendziora, Ecem Z. Ergun, Elke Sattler, Charlotte Gust, Lars E. French, Işın Sinem Bağcı, Daniela Hartmann

**Affiliations:** ^1^ Department of Dermatology and Allergy University Hospital Ludwig Maximilian University Munich Germany; ^2^ PhD School in Clinical and Experimental Medicine University of Modena and Reggio Emilia Modena Italy; ^3^ Istanbul Training and Research Hospital Department of Dermatology Istanbul Turkey; ^4^ Dr. Phillip Frost Department of Dermatology & Cutaneous Surgery Miller School of Medicine, University of Miami Miami Florida USA; ^5^ Department of Dermatology Stanford University Stanford California USA

**Keywords:** moisturizers, noninvasive imaging, optical coherence tomography, Raman spectroscopy, skin hydration

## Abstract

Skin is our barrier against environmental damage. Moisturizers are widely used to increase hydration and barrier integrity of the skin; however, there are contrasting observations on their in vivo effects in real‐life settings. In cosmetic studies, corneometers and tewameters are traditionally used to assess skin hydration. In this study, two novel noninvasive diagnostic techniques, optical coherence tomography (OCT) and confocal Raman spectroscopy, were used to analyze stratum corneum and epidermal thickness (ET), water content, blood flow in function of depth, skin roughness, attenuation coefficient, natural moisturizing factor, ceramides and free fatty acids, cholesterol, urea, and lactates in 20 female subjects aged between 30 and 45 before and after 2 weeks application of a commercially available moisturizing lotion on one forearm. The untreated forearm served as control. A third measurement was conducted 1 week after cessation of moisturizing to verify whether the changes in the analyzed parameters persisted. We noticed a reduction in skin roughness, an increase in ceramides and free fatty acids and a not statistically significant increase in ET. As a conclusion, short time moisturizing appears insufficient to provide significant changes in skin morphology and composition, as assessed by OCT and RS. Novel noninvasive imaging methods are suitable for the evaluation of skin response to topical moisturizers. Further studies on larger sample size and longer treatment schedules are needed to analyze changes under treatment with moisturizers and to standardize the use of novel noninvasive diagnostic techniques.

## 1 INTRODUCTION

Skin has a crucial role as a barrier against environmental factors and must be regularly hydrated for protecting its integrity and function. The outermost layer of the epidermis, the stratum corneum (SC), is primarily responsible for preventing dehydration of underlying tissues. Its complex structure is composed of corneocytes, their hygroscopic material, collectively referred to as natural moisturizing factor (NMF), and an intercellular lipid bilayer matrix. NMF is the degradation product of filaggrin, acting as an efficient humectant and containing amino acids, ceramides, cholesterol, and fatty acids. If SC water content reduces to a critical level, normal desquamation is impaired, leading to the accumulation of corneocytes on the skin surface and causing the appearance of dryness and scaling.^1^

Moisturizers increase skin hydration and re‐establish skin lipid film after cleansing; they can also function as vehicles to deliver active ingredients into the skin.^1^

The growing interest for skin barrier function together with the flourishing cosmetic industry led to an increasing number of studies trying to analyze the short‐ and long‐term effects of moisturizers on dry skin. However, many studies lack standardized methods and devices that can be perfectly reproducible; on the other hand, difficulty in performing invasive biopsies for such studies limits the accuracy of the results.

Noninvasive imaging techniques can be used for a painless, in vivo investigation of skin morphology and physiology, thus avoiding biopsies. Optical coherence tomography (OCT), mainly employed for skin cancer diagnosis, offers two‐dimensional vertical imaging of the skin up to a depth of 1.5–2 mm^2^, ^3^, ^4^, ^5^, ^6^, ^7^, ^8^; it can assess skin thickness with a good correlation with histopathology.^9^ Confocal Raman spectroscopy (CRS), on the other hand, provides information on the molecular composition of the skin including water content, NMF, ceramide, and cholesterol levels. CRS has been successfully used to assess pathophysiological skin conditions and physiological skin parameters.^10^, ^11^, ^12^, ^13^, ^14^, ^15^, ^16^, ^17^, ^18^ In addition, when combined with OCT, it showed a positive correlation in measuring SC thickness.^19^

Even though various studies have been conducted on the effects of moisturizer application on skin, there are little data available assessing the posttreatment effects of moisturizers using novel noninvasive imaging methods.^2^, ^3^, ^19^, ^20^, ^21^, ^22^ The aim of this study was to noninvasively investigate skin changes following the application of a hygroscopic moisturizer over 2 weeks and to follow up its short‐term efficacy 1 week after cessation of application, using OCT and CRS.

## 2 METHODS

### 2.1 Subjects and study design

The noninvasive imaging and treatment were performed on the healthy skin of 20 female volunteers of Caucasian ethnicity (Fitzpatrick skin type 1–3) of 30–45 years of age. Before enrolment, information about the imaging methods and the moisturizing agent (Sebamed Lotion) were given to each subject and written informed consent was obtained. The experimental protocol was approved by the ethics committee of the Ludwig‐Maximilian University of Munich (reference no.: 19–028).

Imaging was performed during three different visits. On day 0 (before treatment), both volar forearms of each subject were examined using OCT and CRS in a standardized manner while the subjects were sitting on a chair facing the examination table in order to avoid positional variations during the imaging. The measurements were taken in the same room at a standard temperature (21–22°C), humidity, and light condition. Measurements were performed on areas without visible skin lesions (such as solar lentigines, melanocytic nevi, and scars) to assess and compare the features of healthy skin. The subjects had been instructed to stop showering, cleaning the forearms and applying creams/lotions on the forearms 24 h prior to the baseline measurement. On day 1, subjects started applying the moisturizer lotion, whose list of ingredients stated on the packaging are aqua, glycerin, cetearyl alcohol, sorbitol, hexyldecanol, hexyldecyl laurate, chamomilla recutita flower extract, allantoin, sodium cetearyl sulfate, citric acid, sodium hydroxide, sodium acetate, sodium ascorbate, xanthan gum, dimethicone, perfume, alcohol, phenoxyethanol, sodium benzoate, and benzyl alcohol, only to the volar side of the left forearm once daily over a period of 2 weeks. The right forearm was not treated in order to serve as the control side. No other topical treatments were allowed on both forearms. On day 15 (24–36 h after the last moisturizer application), volar sides of both forearms were examined with OCT and CRS. The subjects were then asked to leave both forearms untreated and free from any topical treatments for the following 7 days. On day 21, OCT and CRS measurements were performed on both forearms.

### 2.2 Optical coherence tomography

The OCT device used in our study (VivoSight; Michelson Diagnostics, Kent, UK) scans an area of 6 × 6 mm, reaching a penetration depth of 1.5–2 mm with <7.5 μm as a lateral and 5–10 μm as an axial resolution. This multibeam Fourier domain OCT uses a swept‐wavelength diode laser (Axsun, Billerica, USA) operating at a centre wavelength of 1305 nm. A flexible handpiece allows measurements of any skin region. By lateral scanning, it provides two‐dimensional cross‐sectional images of the skin. Once the images are acquired, the system allows two‐dimensional manual measurements of the visualized structures. According to our study protocol, 10 different measurements were performed on the OCT image of each subject and the mean value was recorded. Evaluation of the images was performed in a blinded way. Using the integrated software VivoTools, provided by Michelson Diagnostics, the following skin parameters were automatically retrieved from the acquired images: blood flow (BF) at 0.1 and 0.3 mm depth from the surface, average epidermal thickness (ET), light attenuation coefficient (AC), arithmetic mean skin roughness (Ra), and average depth of skin roughness (Rz) (Figure S2). Capillary BF is quantified by calculating the signal intensity of speckle variance produced by local movement ( = dynamic) at different depths ranging from 0.0 (no vessels) to 1.0 (100% of the tissue is made of vessels). Average ET is obtained by analyzing the average OCT intensity profile. The Ra relates to a smoothing algorithm, detecting variations from the mean measurement of the skin's curvatures; thus, a higher Ra is related to higher skin wrinkling. Analogously, Rz calculates the difference between the maximum and minimum deviations from the mean surface measurement, so that the higher the Rz, the more is the wrinkling. Such measurements can be influenced by the pressure of the probe and are suited for measuring wrinkles interpreted as tissue texture and not as gross mimic wrinkles. Optical AC determines the rate at which light is scattered from the tissue with increasing depth; a strong light scattering as in very dense tissues (dense collagen fibers, keratin) corresponds to bright OCT images and high AC, while a higher light absorption (water, dilated blood vessels, dispersed collagen fibers) corresponds to darker images and low AC. As a consequence, a more hydrated‐moisturized skin has a lower AC and appears darker than dry skin.

### 2.3 Confocal Raman spectroscopy

CRS analyses were performed using a confocal Raman spectrometer (gen2 Skin Composition Analyzer; RiverD International B.V., Rotterdam, The Netherlands) with an inverted confocal microscope containing an oil immersion objective. CRS is vibrational spectroscopy based on the principle of inelastic light scattering. Following the exposure of monochromatic laser light, the energy of the photons is transferred to the molecules of the sample, subsequently originating the scattered light. The amount of the transferred energy depends on the masses of the atoms of the particular molecule and the chemical bonds between them; therefore, the Raman spectrum is highly molecule specific.^23^, ^24^ CRS allows analyzing the molecular composition of skin and their distance to the skin surface with high spatial and temporal resolution. It provides quantitative and semi‐quantitative concentration profiles of compounds in SC such as water content in mass %, NMF, ceramide, and cholesterol.^25^, ^26^ Moreover, it enables monitoring the changes in molecular concentrations of skin compounds following the application of chemicals or drugs.^18^, ^27^

The device has two incorporated lasers, operating at wavelengths of 671 and 785 nm, which are nondestructive for biological tissues. Data are collected by a CCD detector. The experiments were carried out in two different spectral regions: extended fingerprint (400–2000 cm^−1^) and high wavenumber (2000–4000 cm^−1^) regions. The extended fingerprint region uses a diode laser of 785 nm wavelength and with a maximum power at the sample of 28 mW, whereas the high wavenumber region uses a diode laser of 671 nm wavelength and with maximum power at the sample of 20 mW. The area of the laser spot on the samples was 1 μm in diameter. The scanned areas can be directly visualized on the screen.

Fingerprint spectra were recorded from 0 up to a depth of 28 μm, in 4 μm increments, with an exposure time of 5 s. High wavenumber spectra were recorded in two tracks. The first track collected data from 0 up to a depth of 24 μm, in 2 μm increments, with an exposure time of 1 s; the second track collected data from 0 up to a depth of 24 μm, in 4 μm increments, with an exposure time of 1 s, then from 24 μm up to a depth of 48 μm, in 8 μm increments, with an exposure time of 2 s, and from 48 μm up to a depth of 118 μm in 10 μm increments, with an exposure time of 4 s. Eight different locations for each forearm were scanned at every time point. Spectra were obtained by positioning the arm on the fused silica window of the measurement stage of the spectrometer.

Target analyses were water content in the SC and epidermis, NMF, ceramides and free fatty acids, cholesterol, urea, and lactate. Water content at different depths and nonrestricted multiple least‐square fit were calculated semiautomatically by SkinTools software through the default methods “water content” and “NMF.” The software automatically detects and excludes outliers, also providing the option of manually reviewing altered spectra.

The skin water content as an indicator of depth was determined by the ratio of the integrated peak areas of protein keratin (between 2910 and 2960 cm^−1^) and a water molecule (between 3350 and 3550 cm^−1^) in the high wavenumber region (2500−4000 cm^−1^) resulting from CH and OH stretching vibrations, respectively. The detailed procedures were described elsewhere by Caspers et al.^25^ The default method ‘NMF’ uses the fingerprint or low wavenumber region (400−1800 cm−1) to analyze the following spectra in function of their depth: pyrrolidone carboxylic acid, urocanic acid, urea, lactate, alanine, serine, phenylalanine, glycine, ornithine, arginine, citrulline, and histidine. Data are displayed as fit coefficients as a function of depth, which are called NMF profiles.

### 2.4 Data and statistical analysis

Data extrapolation of Raman spectroscopy was performed semi‐automatically using the Skintools 3 software (RiverD International B.V.).

The two‐way analysis of variance (ANOVA) test was used to assess the variables in three measurement timepoints. Paired *t*‐tests were used for pairwise comparisons. Shapiro–Wilk's tests were applied for confirming the assumption of normal distribution in the data. Outliers located higher than 1.5* interquartile range above the upper quartile or lower than 1.5* interquartile range below the lower quartile were capped. *p*‐Values below 0.05 were considered statistically significant. For multiple paired *t*‐tests, *p*‐values were adjusted using the Bonferroni multiple testing correction method. All tests were two‐tailed. R (version 3.6.0, 2019, R Foundation for Statistical Computing) was applied for all statistical calculations. The outliers R package was used for capping outliers,^28^ the ggpubrR package for creating boxplots,^29^ and the rstatix R package for Shapiro–Wilk's tests, two‐way repeated‐measures ANOVA tests, and pairwise *t*‐tests.^30^

## 3 RESULTS

### 3.1 Epidermis thickness manually measured by OCT

**FIGURE 1:**
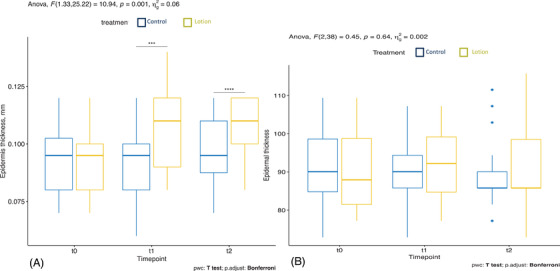
Epidermis thickness manually measured by optical coherence tomography (OCT) (A) and automatically measured by OCT VivoTools software (B) in the treated arm (yellow) and in the control arm (blue)

The control arm had a mean ± SD ET of 0.094 ± 0.014 mm, and the treated arm of 0.094 ± 0.013 mm at baseline. In the treated arm, ET measured by OCT significantly increased from baseline to T1 (0.108 ± 0.016 mm), remaining stable at T2 (0.108 ± 0.012 mm). The control arm did not show a statistically significant change (T1 0.093 ± 0.015 mm, T2 0.096 ± 0.014 mm) (Figure 1A, Figure S1).

### 3.2 Epidermis thickness automatically measured by OCT VivoTools software

The mean ± SD baseline ET of the untreated and treated arms were 91.081 ± 9.901 μm and 90.952 ± 9.395 μm, respectively. In the moisturized arm, ET increased from T0 to T1 (92.212 ± 8.717 μm), however not statistically significant (*p *= 0.569), and decreased at T2 (90.38 ± 10.08 μm). In the control arm, ET decreased at T1 (90.18 ± 9.447 μm), and was further reduced at T2 (88.996 ± 9.004 μm), although not statistically significant (*p *= 0.687) (Figure 1B).

### 3.3 SC thickness measured by CRS

The treated arm had a mean ± SD SC thickness of 15.229 ± 2.568 μm at baseline, decreased to 14.873 ± 2.615 μm at T1 and increased to 15.674 ± 2.208 μm at T2. The changes were not statistically significant.

**FIGURE 2:**
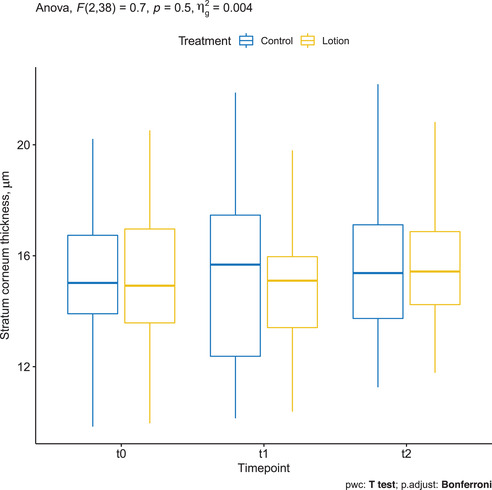
Stratum corneum (SC) thickness measured by confocal Raman spectroscopy (CRS) in the treated arm (yellow) and in the control arm (blue)

In the control arm, the mean ± SD SC thickness was 15.114 ± 2.590 μm at baseline, increased to 15.514 ± 3.575 μm at T1 and 15.575 ± 3.575 μm at T2. The changes were not statistically significant (Figure 2).

### 3.4 Ceramides and free fatty acids assessed by CRS

Mean ± SD ceramide and free fatty acids concentration was 0.040 ± 0.005 at baseline in the treatment group, which slightly increased to 0.043 ± 0.007 at T1, and decreased after stopping treatment to 0.038 ± 0.007 at T2.

On the other hand, mean ± SD ceramide and free fatty acids concentration in controls was 0.042 ± 0.006 at baseline, reduced to 0.039 ± 0.006 at T1, and remained stable at T2.

**FIGURE 3:**
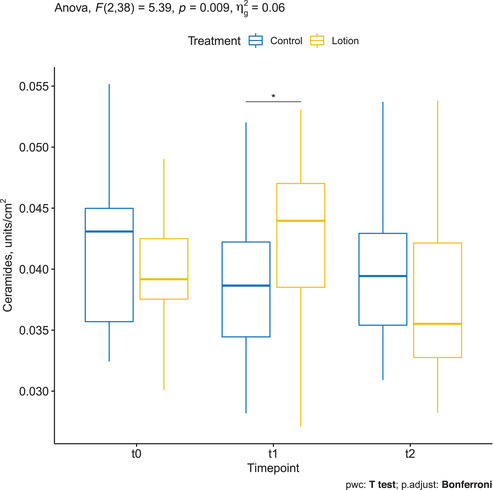
Ceramides and free fatty acids assessed by confocal Raman spectroscopy (CRS) in the treated arm (yellow) and in the control arm (blue)

The two‐way repeated measures ANOVA showed a statistically significant two‐way interaction between treatment and time, *F*(2, 38) = 5.4, *p* = 0.009. Pairwise comparisons between treatment groups showed a statistically significant difference between the treatment and control group at T1 (*t*(19) = −2.1, *p* = 0.046) but not at T0 (*t*(19) = 1.6, *p* = 0.116) and T2 (*t*(19) = 0.8, *p* = 0.430) (Figure 3).

### 3.5 NMF assessed by CRS

**FIGURE 4:**
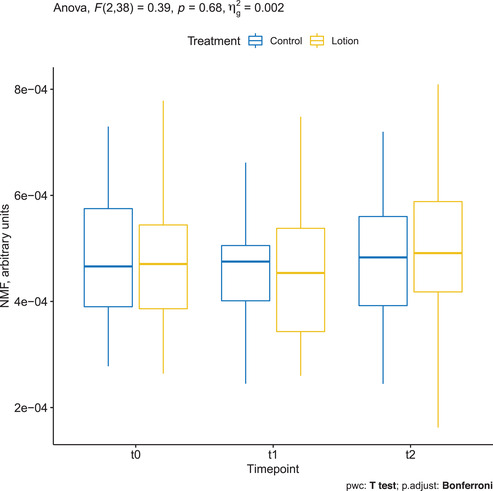
Natural moisturizing factor (NMF) assessed by confocal Raman spectroscopy (CRS) in the treated arm (yellow) and in the control arm (blue)

Mean ± SD NMF of the treatment group was 0.000481 ± 0.000138 at baseline, decreased to 0.000459 ± 0.000135 at T1, and increased to 0.000499 ± 0.000133 at T2. The changes were not statistically significant (*p* = 0.68). Mean ± SD NMF of the control group was 0.000480 ± 0.000132 at baseline, decreased to 0.000470 ± 0.000124 at T1, and increased to 0.000484 ± 0.000129 at T2. The changes were not statistically significant (*p* = 0.8) (Figure 4).

### 3.6 Cholesterol assessed by CRS

**FIGURE 5:**
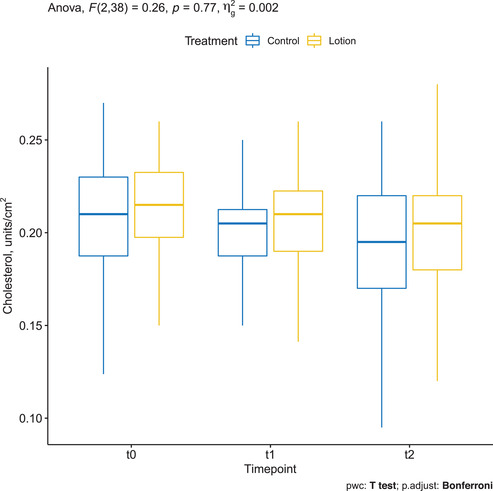
Cholesterol assessed by confocal Raman spectroscopy (CRS) in the treated arm (yellow) and in the control arm (blue)

Mean ± SD cholesterol was 0.212 ± 0.030 at baseline in the treatment group and decreased to 0.206 ± 0.031 at T1 and 0.203 ± 0.037 at T2. The changes were not statistically significant (*p* = 0.77). Mean ± SD cholesterol was 0.203 ± 0.044 at baseline in the control group and decreased to 0.200 ± 0.031 at T1 and 0.188 ± 0.048 at T2. The changes were not statistically significant (Figure 5).

### 3.7 Urea assessed by CRS

**FIGURE 6:**
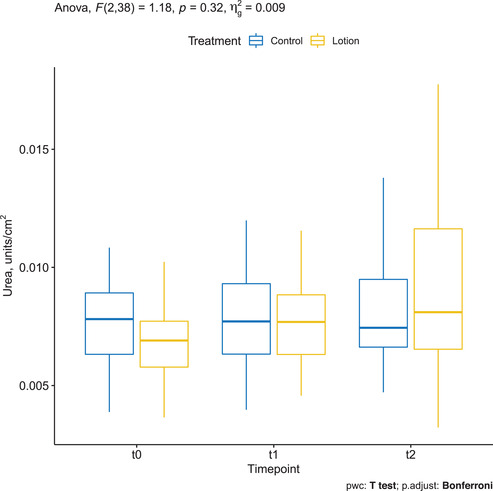
Urea assessed by confocal Raman spectroscopy (CRS) in the treated arm (yellow) and in the control arm (blue)

Mean ± SD urea was 0.007 ± 0.002 at baseline in the treatment group and increased to 0.008 ± 0.002 at T1 and 0.009 ± 0.003 at T2. The changes were not statistically significant (*p* = 0.32). Mean ± SD urea was 0.008 ± 0.002 at baseline in the control group, remained stable to 0.008 ± 0.002 at T1, and increased to 0.009 ± 0.003 at T2. The changes were not statistically significant (Figure 6).

### 3.8 Lactate assessed by CRS

Mean ± SD lactate was 0.009 ± 0.002 at baseline in the treatment group and increased to 0.012 ± 0.004 at T1 and 0.010 ± 0.005 at T2.

Mean ± SD lactate was 0.010 ± 0.002 at baseline in controls and remained stable to 0.010 ± 0.005 at T1 and 0.010 ± 0.004 at T2.

**FIGURE 7:**
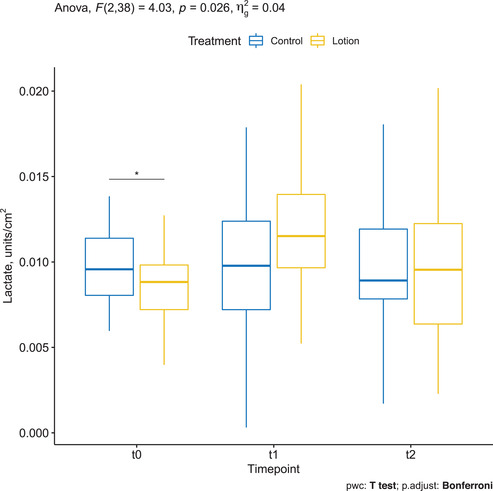
Lactate assessed by confocal Raman spectroscopy (CRS) in the treated arm (yellow) and in the control arm (blue)

The two‐way repeated measures ANOVA showed a statistically significant two‐way interaction between treatment and time (*p* = 0.026). Pairwise comparisons between time points showed a statistically significant difference in the treatment group between T0 and T1 (*p* = 0.0008), but not between T1 and T2 (*p* = 0.079) (Figure 7).

### 3.9 Water content assessed by CRS

**FIGURE 8:**
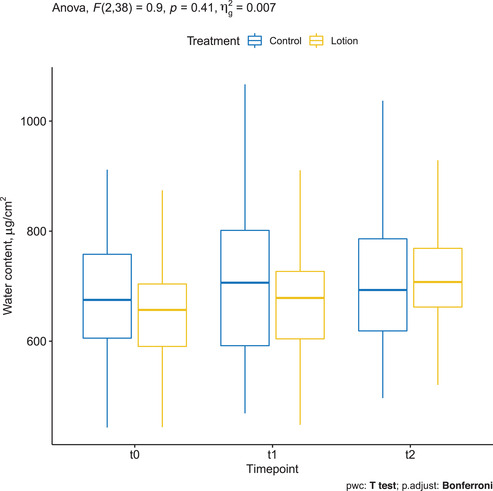
Water content assessed by confocal Raman spectroscopy (CRS) in the treated arm (yellow) and in the control arm (blue)

Mean ± SD water content measured up to 118 μm depth on arms treated with the moisturizing lotion was 662.212 ± 105.494 μg/cm^2^ at baseline, increased to 667.829 ± 125.928 at T1, and further increased to 720.424 ± 106.053 at T2. Changes were, however, not statistically significant (*p* = 0.41). Mean ± SD water content measured up to 118 μm depth on control arms was 674.337 ± 120.843 μg/cm^2^ at baseline, increased to 704.241 ± 158.182 at T1, and further increased to 708.245 ± 126.438 at T2. Changes were, however, not statistically significant (Figure 8).

### 3.10 Blood flow at 0.1 mm depth measured by OCT VivoTools software

**FIGURE 9:**
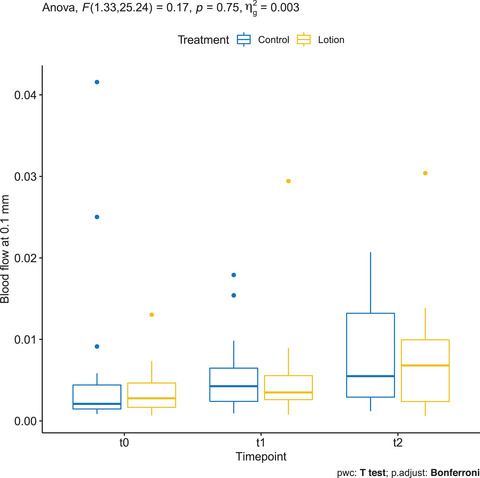
Blood flow at 0.1 mm depth measured by OCT VivoTools software in the treated arm (yellow) and in the control arm (blue)

Mean ± SD baseline BF in the treatment and control groups was 0.006 ± 0.01 and 0.006 ± 0.005, respectively. At T1, it was 0.006 ± 0.005 in the control group and 0.008 ± 0.007 in the treatment group. At T2, it was 0.008 ± 0.007 in both treated and untreated arms, and the difference was statistically significant only in the treated arm (*p* = 0.004) (Figure 9).

### 3.11 Blood flow at 0.3 mm depth measured by OCT VivoTools software

**FIGURE 10:**
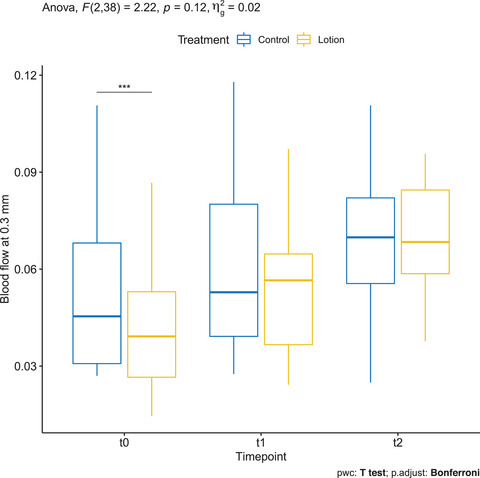
Blood flow at 0.3 mm depth measured by OCT VivoTools software in the treated arm (yellow) and in the control arm (blue)

Mean ± SD baseline BF of the control and treatment groups was 0.053 ± 0.026 and 0.04 ± 0.019. respectively. At T1, it was 0.059 ± 0.024 in the control group and 0.054 ± 0.022 in the treatment group. At T2, it was 0.067 ± 0.022 in the control group and 0.068 ± 0.018 in the treatment group; the differences between timepoints were statistically significant only in the treated arm (*p* < 0.05) (Figure 10).

### 3.12 Skin roughness (Ra) measured by OCT VivoTools software

**FIGURE 11:**
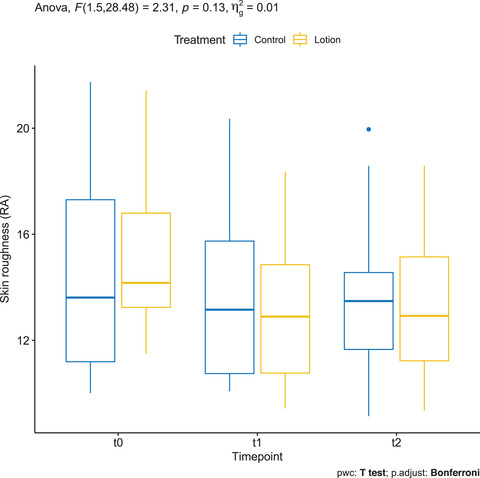
Skin roughness (Ra) measured by OCT VivoTools software in the treated arm (yellow) and in the control arm (blue)

Mean ± SD Ra of the treatment group in T0, T1, and T2 was 15.457 ± 3.311, 13.061 ± 2.784, and 13.248 ± 2.579, respectively. On the other hand, Ra in the controls in T0, T1, and T2 was 14.505 ± 3.973, 13.9 ± 3.214, and 13.677 ± 2.395, respectively. There was a statistically significant decrease between baseline and T1 only in the treated arm (*p* = 0.01) (Figure 11, Figure S2).

### 3.13 Skin roughness (Rz) measured by OCT VivoTools software

**FIGURE 12:**
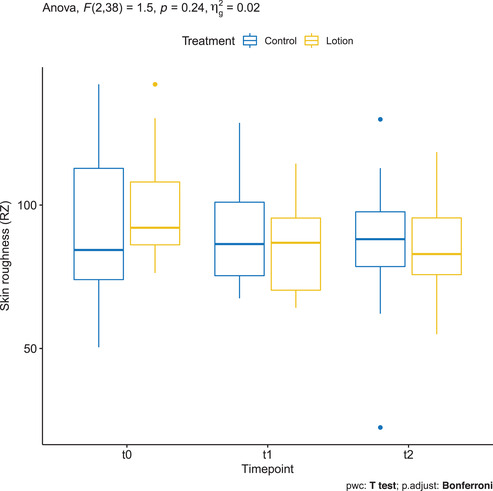
Skin roughness (Rz) measured by OCT VivoTools software in the treated arm (yellow) and in the control arm (blue)

Mean ± SD Rz of the treatment group in T0, T1, and T2 was 98.641 ± 18.532, 85.378 ± 14.979, and 85.763 ± 16.493, respectively. On the other hand, Rz in the controls in T0, T1 and T2 was 92.766 ± 25.653, 91.516 ± 19.593, and 86.406 ± 22.552, respectively. There was a statistically significant decrease between baseline and T1 only in the treated arm (*p* = 0.01) (Figure 12, Figure S2).

### 3.14 Attenuation coefficient measured by OCT VivoTools software

**FIGURE 13:**
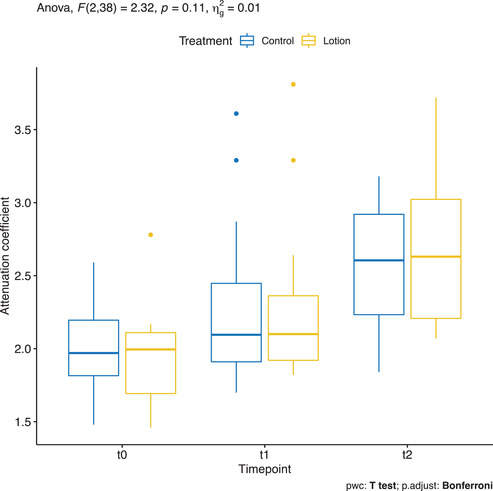
Attenuation coefficient measured by OCT VivoTools software in the treated arm (yellow) and in the control arm (blue)

Mean ± SD AC in the untreated arm was 1.996 ± 0.291, in the moisturized arm of 1.942 ± 0.303 at baseline. AC increased, however, in both untreated and moisturized arms from baseline to T1 (2.24 ± 0.506 and 2.262 ± 0.514, respectively) and from T1 to T2 (2.566 ± 0.43 and 2.721± 0.544) so that there was no statistical significance in the group comparison (Figure 13).

## 4 DISCUSSION

The integrity of the skin is needed to preserve water in the epidermis, which is essential for hydrolytic enzymatic reactions in keratinocytes and maintaining the defence function against microorganisms, chemicals, and mechanical stress.

Moisturizers can be used to support the natural skin barrier. They act through two basic mechanisms: occlusion and humectancy. Occlusive moisturizers function as a water‐impermeable barrier over the skin surface, thereby preventing Transepidermal water loss (TEWL) and creating an optimal environment for restoration of the SC barrier. Rehydration of SC occurs by water that is attracted from the deeper epidermal and dermal tissues. Humectants are substances that attract moisture from the environment. They also induce corneocyte swelling and diminish voids, leading to the improvement of dry skin.^4^,^31^, ^32^ In addition, moisturizers change the mRNA expression of genes essential for keratinocyte differentiation and desquamation thereby modify the skin barrier function.^11^ Although moisturizers have been recommended by dermatologists over the years, some studies proved that they can sometimes weaken the skin barrier function and increase the susceptibility of the skin to irritants.^7^, ^8^, ^13^, ^14^, ^15^, ^16^ Moreover, the hydrating efficacy depends on the type of moisturizer and the treated subject.^12^ Hence, additional studies are required to improve our understanding of the topic.

Most studies measuring skin hydration have used equipment that relies on changes in electrical properties, while alterations in skin barrier function are typically measured by TEWL. Those methods are quick and painless; however, they are not fully standardized, still deeply influenced by multiple variables such as humidity and room temperature, and do not provide any actual quantitative indication of water distribution, epidermal and SC thickness, and NMF components. Novel noninvasive diagnostic technologies offer a more precise and standardized assessment of skin. Reflectance confocal microscopy (RCM) provides high‐resolution horizontal imaging of epidermis and papillary dermis enabling assessment of cellular details. In our previous study assessing the same moisturizing lotion in different ethnic skin types, we observed a reduced width of skin folds following 2 weeks application of the same moisturizing lotion on the treated arms as compared to the control arms using RCM.^33^ On the other hand, Manfredini et al. showed reduced micro‐scaling and epidermal irregularity, as well as higher interkeratinocytes' brightness with RCM following moisturizer application.^21^ These results might be compatible with the decrease in SC thickness leading to less scaling of the skin surface.

Recently, a new user‐friendly and semi‐automated CRS was able to determine in vivo real‐time skin water profiles in the function of depth, estimate SC thickness, and the effect of moisturizers on the skin by combining the principle of confocal microscopy with Raman spectroscopy.^18^ A study that measured the effects of moisturizers and compared CRS and OCT obtained a positive correlation between these two methods in measuring SC thickness.^19^ More recently, CRS alone has been used to assess the efficacy of moisturizers on skin hydration in 12 patients; the authors of this pilot study compared water and NMF contents of skin after the application of commercial moisturizer products and stated that different products do not hydrate the skin to the same level, and that some of them might even dehydrate the skin, depending on the formulations.^18^

If we focus on OCT, the device was reported to be an efficient tool for the characterization of skin morphology, also compared to fluorescence laser scanning microscopy and conventional light microscopy.^15^, ^22^, ^34^, ^35^ In a recent review, Kislevitz et al. highlighted the role of OCT in aesthetic skin assessment, not only for the skin surface but also for the underlying epidermis and dermis. Thanks to the new automated software SkinTools in fact, it is possible to take semi‐automated and objective measurements of various skin parameters, such as BF at different depths, which is particularly useful for measuring vasodilation and neoangiogenesis,^34^ ET, AC, and Ra/Rz.

In this study, a moisturizing lotion was applied on one arm for 2 weeks. Even though emollients can be irritating at high concentrations, no adverse events were reported in our study. We found an increase in the ET of the treated subjects using OCT. While the manual measurements revealed a significant increase in ET on the treated arm, the semi‐automatic calculation of ET displayed a nonstatistically significant change. This aspect sheds light on the limited accuracy of repeated manual measurements in determining ET compared to more standardized, semiautomatic, or automatic measurements extrapolated by dedicated software. The thickness of SC remained unchanged after moisturizer application, as extensively confirmed by CRS^19^ (Figure 2).

In our previous study,^2^ we also observed an increase in the ET by OCT by manual measurements following 2‐week application schedule, and hypothesized a deeper and enhanced penetration of hygroscopic lotions. In this study, we aimed to verify the above‐mentioned hypothesis and detect the distribution of volume increase among skin compartments through the standardized and objective measures of CRS. In fact, we demonstrated that the thickness of SC did not change significantly at the different time points analyzed, but showed a trend toward reduction at T1 in the treatment arm, which is against the common belief the moisturizers accumulate in and causes the swelling of SC.^36^, ^37^ Such a reduction of SC thickness after short‐term treatment was also observed in another study with CRS.^19^ This might be due to the increased exfoliation of the superficial SC produced by the mechanical action of the regular moisturizer application.

On the other hand, we found an increase in the ET following moisturizer application, whereas control side ET gradually decreased. This supports our hypothesis that the moisturizing lotion mainly affected the deeper epidermal layers rather than the uppermost SC. Parallel to the ET, a positive trend of the water content in the treatment arm could be measured by CRS. However, the changes in ET and water content did not show a statistical, which might be due to the small sample size, interpersonal variability and the short treatment period. Tippavajhala et al. hypothesized that long treatment duration (over 30 days) could show better results in improving skin hydration.^18^

NMF showed a negative trend from baseline to T1, and we observed a positive trend from T1 to T2 (after suspending treatment). This is in line with previous findings showing a reduction of NMF after treatment with moisturizers, following the hypothesis that lotions can temporarily interfere with the functional organization of SC and composition of the skin lipid film.^18^

The ceramide and free fatty acids content in the skin showed a statistically significant improvement from baseline to T1 (during daily application of the moisturizer), which decreased after stopping treatment. Ceramides are produced in stratum granulosum and transported to SC and constitute almost 50% of the total SC volume.^38^

Although we showed a diminished SC following moisturizing, an induction of ceramide production by the ingredients of the moisturizer could be speculated. This result also supports the hypothesis that regular moisturizer application improves the skin barrier function.

Concerning skin morphology and blood vessels analysis, we reported an increase in BF at a depth of 0.3 mm between timelines T1 and T2 in the treated arm, probably due to mechanical stimulation causing vasodilatation. Accordingly, we saw a significant decrease in the skin roughness parameters (Ra and Rz) between baseline and T1 only in the moisturized arm, indicating a smoothening of the skin surface, in line with our previous study.^33^ We found no statistically significant changes in the AC; in particular, we could not observe the expected decrease of AC observed in other studies after treatment with glycerol, probably because our measurements were not taken immediately after moisturizing.^39^

In conclusion, we observed an increase in ceramides and free fatty acids and a decrease in skin roughness indicating a temporary swelling of lower epidermal compartments (thus, smoothening of the skin surface) under treatment with a moisturizing lotion; we observed a tendency to increased ET which could not reach statistical significance. However, novel noninvasive diagnostic methods such as OCT and CRS are suitable for monitoring the effects of moisturizers in vivo, and they can be combined to observe morphological and molecular perspectives. Further studies on larger sample size and longer treatment schedule could be helpful to better standardize the use of the above‐mentioned tools in cosmetic studies and better characterize the skin changes.

## CONFLICT OF INTEREST

The authors declare no conflict of interest.

## Supporting information

SUPPORTING INFORMATION
