# Supplementary material for: In vivo examination of healthy human skin after short‐time treatment with moisturizers using confocal Raman spectroscopy and optical coherence tomography: Preliminary observations
Source: Skin Res Technol. 2021 Sep 23;28(1):119–32. doi: 10.1111/srt.13101 (PMC9907652; doi:10.1111/srt.13101)

- **Supplementary figure**
- 1. Optical coherence tomography images of the left forearm (treated) at baseline (A-C), T1 (D-F) and T2 (G-I) . In the 3D reconstruction (A,D,G), the skin folds are visible. These appear flattened at T1 (D) compared to baseline (A), with increasing hydration. The same effect can be observed in the en-face images (B, E, H), together with hair and hair follicles. In vertical mode, the uppermost layer corresponds to the Stratum corneum (SC) (asterisk), while the dermo-epidermal junction (DEJ) is marked by an arrow.


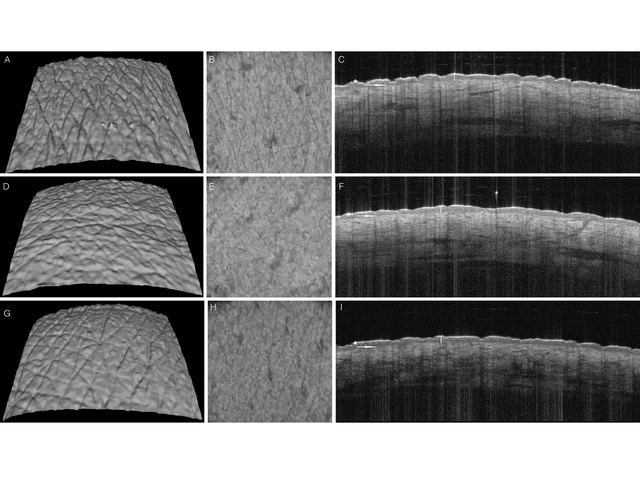


- 2. Measurement of skin roughness using OCT VivoTools software®: the roughness tool also produces a graphical image of the skin surface in which the surface shape is exaggerated and colour-coded by height. Ra: mean variation of the surface height; Rq: root-mean-square variation of the surface height; Rz: peak-to-trough difference of the surface height at the lowest and highest points
-
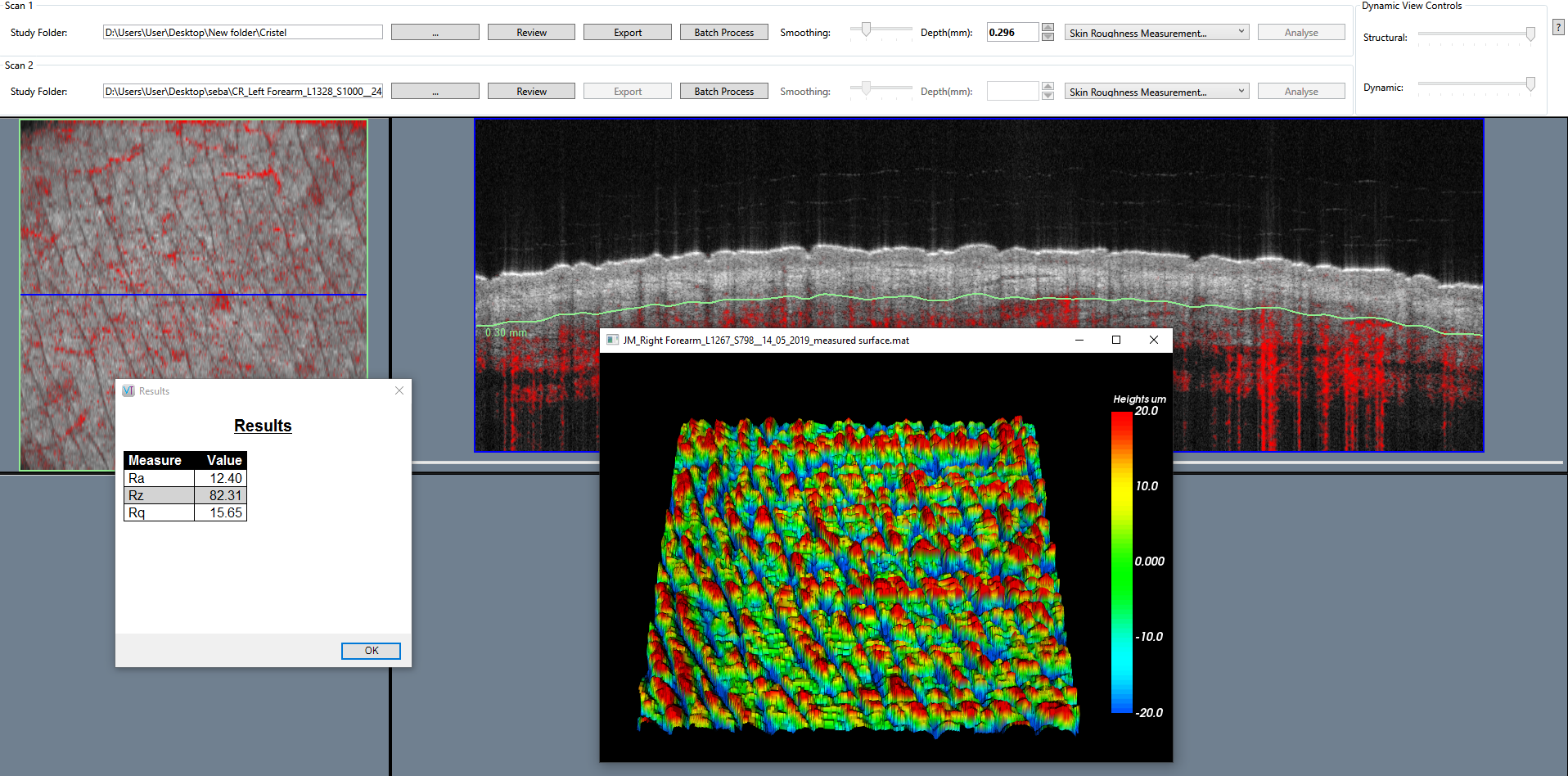

Supplement: Supplementary file 1 — SUPPORTING INFORMATION [file SRT-28-119-s001.docx]
